# Financial barriers and inequalities in healthcare access across East Africa: evidence from demographic and health surveys

**DOI:** 10.3389/frph.2025.1730560

**Published:** 2026-01-15

**Authors:** Omer Adam Farih, Saeed Hassan Mohamed, Asma Mahamoud Abdillahi, Abdirizak Hassan Abokor, Mustafe Abdillahi Ali, Abdisalam Hassan Muse, Hodo Abdikarim

**Affiliations:** 1 Research and Innovation Center, Amoud University, Borama, Somalia; 2 RAIS Analytics, Hargeisa, Somalia; 3 Institute of Social Science, Selçuk University, Konya, Türkiye

**Keywords:** demographic and health surveys (DHS), East Africa, financial barriers, healthcare access, socioeconomic inequality, universal health coverage

## Abstract

**Background:**

Financial barriers are a critical impediment to achieving Universal Health Coverage (UHC), particularly in sub-Saharan Africa. In East Africa, high out-of-pocket health expenditures persist, potentially exacerbating inequities in healthcare access, especially for vulnerable groups like women of reproductive age. This study aimed to assess the prevalence and socioeconomic inequalities of financial barriers to healthcare access among women in eight East African countries.

**Methods:**

We conducted a cross-sectional analysis of nationally representative Demographic and Health Surveys (DHS) from Burundi, Ethiopia, Kenya, Rwanda, Somalia, Somaliland, Tanzania, and Uganda (2016–2022), comprising a weighted sample of 108,175 women. The outcome variable was a self-reported big problem with “money needed for treatment.” We performed descriptive statistics, calculated concentration indices to measure economic inequality, and used a multivariable multilevel binary logistic regression to identify associated factors.

**Results:**

Nearly half (49.7%) of the women reported financial barriers, with significant cross-country variation, ranging from 64.8% in Somalia to 36.2% in Tanzania. Financial hardship was disproportionately concentrated among poorer economic groups, as evidenced by negative concentration indices across all countries (e.g., Rwanda: −0.0825; Ethiopia: −0.0737). Multilevel analysis revealed that lower wealth quintile (AOR=0.21 for richest vs. poorest), no formal education (AOR=0.41 for higher vs. no education), and lack of a bank account (AOR=0.69) were strongly associated with higher odds of financial barriers. A key finding was the reversal of the rural-urban disparity upon adjusting for socioeconomic confounders, suggesting that poverty, not rurality itself, is the primary factor associated with financial access problems.

**Conclusion:**

Financial barriers are the most prevalent and inequitable obstacle to healthcare access for women in East Africa, disproportionately affecting the poor, less educated, and financially excluded. Accelerating progress toward UHC requires health financing reforms that reduce out-of-pocket payments, alongside multi-sectoral policies that address underlying socioeconomic disadvantages through pro-poor interventions and financial inclusion. This focus is justified given their heightened need for maternal, sexual, and reproductive healthcare, and their heightened vulnerability to financial exclusion and catastrophic health expenditures.

## Background

Healthcare services must be accessible, available, affordable, and culturally appropriate for all individuals, a principle that is especially crucial for vulnerable populations such as pregnant women and their infants, who have specific healthcare requirements (1, 2). The concept of “access” is most effectively defined by three interconnected dimensions: availability, affordability, and acceptability (3). Genuine access to healthcare exists when services are present in adequate supply and when populations have a realistic opportunity to use them; barriers emerge from financial, organizational, social, or cultural strains within a community (4). Furthermore, access to comprehensive, high-quality healthcare is essential for health promotion, disability reduction, disease prevention and management, and the advancement of health equity (5). This access is a fundamental human right and a central objective of the global development agenda, explicitly articulated in Sustainable Development Goal (SDG) 3, which aims to ensure healthy lives and promote well-being for all ages by 2030 (6). The core mechanism for this goal is Universal Health Coverage (UHC), which aims to guarantee that all people can access the quality health services they need without suffering financial hardship debate (7).

Current data reveal significant gaps in achieving this access. According to the World Health Organization and the World Bank, approximately 50% of the global population cannot obtain essential health services, and 100 million people are pushed into extreme poverty each year because of health expenses (8). The situation is particularly severe in sub-Saharan Africa, where the level of access to healthcare is notably low at 42.56% (9). Research identifies a multitude of barriers to access, which include financial constraints, geographical location, the education level of the mother and her husband, health insurance status, marital status, place of residence, stigma, and the number of children a woman has (10–13). The consequences of these barriers are profound; the World Bank estimates that 74% of maternal deaths could be prevented with full access to interventions for pregnancy and childbirth complications, particularly emergency obstetric care (14).

The global burden of maternal mortality remains high. In 2015, 302,000 maternal deaths occurred worldwide, with 99% of these deaths taking place in developing regions. Sub-Saharan Africa alone accounted for 66% (201,000) of these deaths, followed by Southern Asia (66,000) (15). The majority of these deaths are preventable through improved access to quality medical care during pregnancy, childbirth, and the postnatal period (16). A persistent and concerning trend in developing countries, particularly in sub-Saharan Africa, is that maternal mortality rates remain high and have in some cases increased despite health initiatives. This challenge is most acute in sub-Saharan Africa, where a large proportion of deliveries occur without the assistance of skilled health personnel (17, 18). More recent data from 2020 shows that approximately 287,000 women still died from preventable pregnancy and childbirth-related causes globally (19). These deaths are not evenly distributed, with the highest rates concentrated in low and lower-middle income countries (LLMICs) (19).

Although progress has been made—the global Maternal Mortality Ratio (MMR) fell by 34% from 339 to 223 deaths per 100,000 live births between 2000 and 2020, and East Africa achieved a 54% reduction (from 756 to 351 per 100,000 live births) in the same period—the pace of decline is insufficient (19). Achieving the SDG target of an MMR below 70 requires an average annual reduction rate (ARR) of 5.5%. However, the global average ARR between 2000 and 2020 was only 2.1%, far short of the 11.6% annual rate needed from 2021 to 2030 to meet the target (20). In response, various health interventions, including specific financing strategies for maternal health services, have been implemented to reduce maternal mortality (21).

Health financing is a core function of health systems and a critical enabler of progress toward UHC (5). Advancing UHC depends on building robust health systems with sustainable financing, which countries pursue through various context-specific approaches (22). Achieving UHC service coverage targets demands a significant increase in healthcare expenditure; for example, reaching the 80% coverage target requires much greater investment (23). Substantial and sustained public budgeting for the health sector is a prerequisite for the health financing reforms needed to advance UHC (24). Countries that have made significant strides toward UHC, such as Thailand, Turkey, Vietnam, and Mexico, did so in part by dramatically increasing their levels of public health funding (25).

Despite the global commitment to UHC, a large segment of the world's population, particularly in low- and middle-income countries (LMICs), continues to lack essential health services. Sub-Saharan Africa (SSA) reports the lowest global rates of healthcare access and service utilization, a problem largely attributable to systemic underfunding and entrenched financial and geographical barriers (26). While East African nations have demonstrated notable progress in improving health indicators like the MMR, the region still struggles with profound inequities in access (27). This lack of equitable access is a major contributor to poor health outcomes, including the fact that SSA is responsible for approximately two-thirds of all global maternal deaths (28). A key factor associated with adverse outcomes like maternal mortality is the lack of timely access to essential health services for women of reproductive age (29).

Among all barriers, financial obstacles are one of the most critical impediments to achieving UHC and health equity in East Africa (21). Many countries in the region, including Kenya, depend heavily on out-of-pocket (OOP) health payments. This financing model is inherently inequitable, frequently causing financial hardship for households and leading to the underuse of necessary health services. Healthcare financing typically involves funds from multiple sources, such as government budgets, targeted government contributions, subsidies, taxes, and foreign grants, all aimed at ensuring sustainable and accessible services (30). The level of investment varies drastically: high-income countries allocate about 14% of total government spending to health, while low-income countries spend an average of only USD $40 per person, representing a mere 4%–8% of total government expenditure (31). These low-income countries face the dual challenge of a high demand for essential services and a constrained ability to mobilize adequate funding, a situation exacerbated by high rates of out-of-pocket health expenditure (OOPHE) (32, 33).

These financial barriers to healthcare access manifest in several ways: direct costs for services and medications; indirect and non-medical costs such as high transportation expenses, long travel times, and lost income from missed work; and the additional burden of informal, unauthorized payments to healthcare providers (34–37). It is critical to note that these barriers are not distributed equally across populations. Studies consistently show that difficulties in accessing healthcare are concentrated among individuals with poor socioeconomic status. This socioeconomic inequality is subsequently linked to significant health disparities, associated with a reduced ability of vulnerable groups, such as poor women or those in rural areas, to afford essential care like regular check-ups, necessary diagnostic tests, and transportation to health facilities (38–40).

Addressing these complex barriers effectively requires policymakers to have access to robust and standardized data on health service utilization and access. The Demographic and Health Surveys (DHS) program provides a crucial and harmonized source of such data, enabling detailed investigation of these issues across multiple East African countries. While other barriers—such as distance to facilities and the need for permission to seek care—remain significant in specific contexts and warrant targeted interventions, the pervasive nature of financial constraints underscores that affordability is a foundational challenge. Addressing financial barriers is thus a critical and necessary component of a comprehensive, multi-pronged strategy to achieve equitable healthcare access.

Therefore, this study aimed to address the following research questions using recent DHS data from eight East African countries: (1) What is the pooled and country-specific prevalence of financial barriers to healthcare access among women of reproductive age? (2) What is the magnitude and direction of socioeconomic inequality in financial barriers across these countries? (3) Which individual, household, and contextual factors are most strongly associated with experiencing financial barriers to healthcare?

## Method

### Data

This study utilized nationally representative household survey data from the Demographic and Health Surveys (DHS) program, a key source for comparable health indicators in low- and middle-income countries. Following the established DHS methodology, which employs a stratified, multi-stage random sampling design to ensure representativeness, we pooled individual women's datasets (IR files) from eight East African countries. These eight countries were selected because they share similar socioeconomic and health system challenges within East Africa, have recent DHS data (2016–2022) ensuring temporal relevance, and allow for meaningful regional comparison. Neighboring countries (Djibouti, South Sudan, Eritrea) were excluded due to outdated or unavailable DHS datasets. The DHS program conducts surveys in each country approximately every 5 years. The years 2016, 2020, and 2022 reflect the most recent survey rounds available for the included countries at the time of analysis. Data for 2017, 2018, 2019, and 2021 were not available because those years did not correspond to scheduled DHS surveys in the studied countries. We pooled individual women's datasets (IR files) from each country by appending them into a single analytic file, with a country identifier variable created for multilevel and fixed-effects modeling. To ensure that the pooled sample remained representative and that no single country disproportionately influenced the estimates due to its sample size, we rescaled the DHS sampling weights. The final weight for each observation was calculated by first normalizing the original DHS weight within each country and then adjusting for each country's relative population size based on United Nations population estimates for women of reproductive age. All analyses—including pooled prevalence estimates, concentration indices, and multilevel regressions—were conducted using Stata's survey (svy) commands, accounting for stratification, clustering, and the rescaled weights to respect the complex sampling design of each DHS. As detailed in Table 1, the analysis incorporated recent DHS rounds (2016–2022) and a final weighted sample of 108,175 women of reproductive age.

**Table 1:** Demographic health surveys.

East African countries	DHS year	Study participants
Burundi	2016	17,269
Ethiopia	2016	15,683
Kenya	2022	16,716
Rwanda	2020	14,634
Somalia	2020	6,633
Somaliland	2020	3,480
Tanzania	2022	15,254
Uganda	2016	18,506
Total sample size		108,175

### Variables

The outcome variable for this study is a self-reported measure of perceived financial difficulty. While it does not capture objective metrics like the exact amount of out-of-pocket expenditure or catastrophic health spending, it serves as a crucial indicator of *perceived affordability* from the user's perspective. This perception is a key determinant of healthcare-seeking behavior, as individuals act based on their own assessment of financial constraints. The use of this binary measure is well-established in health access research using DHS data and allows for consistent cross-country comparisons. However, its subjective nature means it reflects a respondent's personal threshold for what constitutes a “big problem,” and it may not capture more granular levels of financial hardship.

Independent variables included geographical factors: country (Burundi, Ethiopia, Kenya, Rwanda, Somalia, Somaliland, Tanzania, and Uganda) and place of residence (urban or rural). Individual-level factors related to women included age (15–19, 20–24, 25–29, 30–34, 35–39, 40–44, 45–49 years), education level (no education, primary, secondary, higher), marital status (never married, married/living with partner, widowed, divorced/separated), employment status (yes/no), use of mobile money for financial transactions (yes/no), and ownership of a bank or other financial account (yes/no). Household-level socioeconomic status was measured using the wealth index, categorized as poorest, poorer, middle, richer, and richest. These variables were selected to capture both individual and contextual factors associated with financial barriers to healthcare access across East African countries.

### Statistical analysis

All analyses were conducted using Stata 17. Survey weighting was applied separately for each country to account for the complex sampling design of the DHS. Prior to merging datasets, data cleaning was performed, and a country identifier variable was created for each dataset. To ensure comparability, nomadic and rural residence categories for Somalia and Somaliland were combined to match the two-level (urban/rural) structure used in other countries. Marital status categories were also merged to reflect cultural and religious contexts, resulting in the following groups: never married, married/living with partner, and divorced/separated/widowed. Missing values for mobile money usage were recoded as “No” for respondents reported not to own a mobile phone, consistent with DHS coding guidelines.

For the regression analyses, we performed a complete-case analysis. Observations with missing values on the outcome variable (financial barrier) or any independent variable were excluded from the multivariable analysis. Less than 2% of the weighted sample was excluded due to missingness. No other imputation methods were applied.

Descriptive analyses first estimated the proportions of healthcare access barriers with standard errors and 95% confidence intervals. Univariate analyses summarized the distribution of independent variables using frequencies and percentages, followed by bivariate analyses to examine the prevalence of financial barriers across levels of each independent variable. Multicollinearity among independent variables was assessed using the variance inflation factor (VIF). All predictors had VIF values below 2 (range: 1.06–1.69), indicating no serious multicollinearity.

A three-level multilevel binary logistic regression model was specified to account for the hierarchical structure of the data, where women (level 1) are nested within households (level 2), and households are nested within primary sampling units (PSUs) or clusters (level 3). Random intercepts were included at both the household and cluster levels to capture unobserved heterogeneity at these higher levels. Country was included as a fixed effect rather than as an additional random level for two reasons: first, with only eight countries, estimating a country-level variance would be limited by degrees of freedom; and second, our analytical interest included quantifying and comparing specific adjusted differences between these national contexts.

Four sequential models were fitted: Model 0 (empty model) included only the dependent variable; Model I added individual and household factors; Model II added contextual factors (country and place of residence); and Model III included all variables. Model fit and variance explained were evaluated using Akaike Information Criterion (AIC), Bayesian Information Criterion (BIC), log-likelihood, variance components, and intra-class correlation (ICC).

In addition, socioeconomic inequality in financial barriers was assessed using the concentration index (CI), calculated with the cixr command in Stata. All visualizations were generated using R Studio. A significance level of *p* < 0.05 was maintained throughout the analysis.

## Results

### Healthcare access barriers

**Figure 1:**
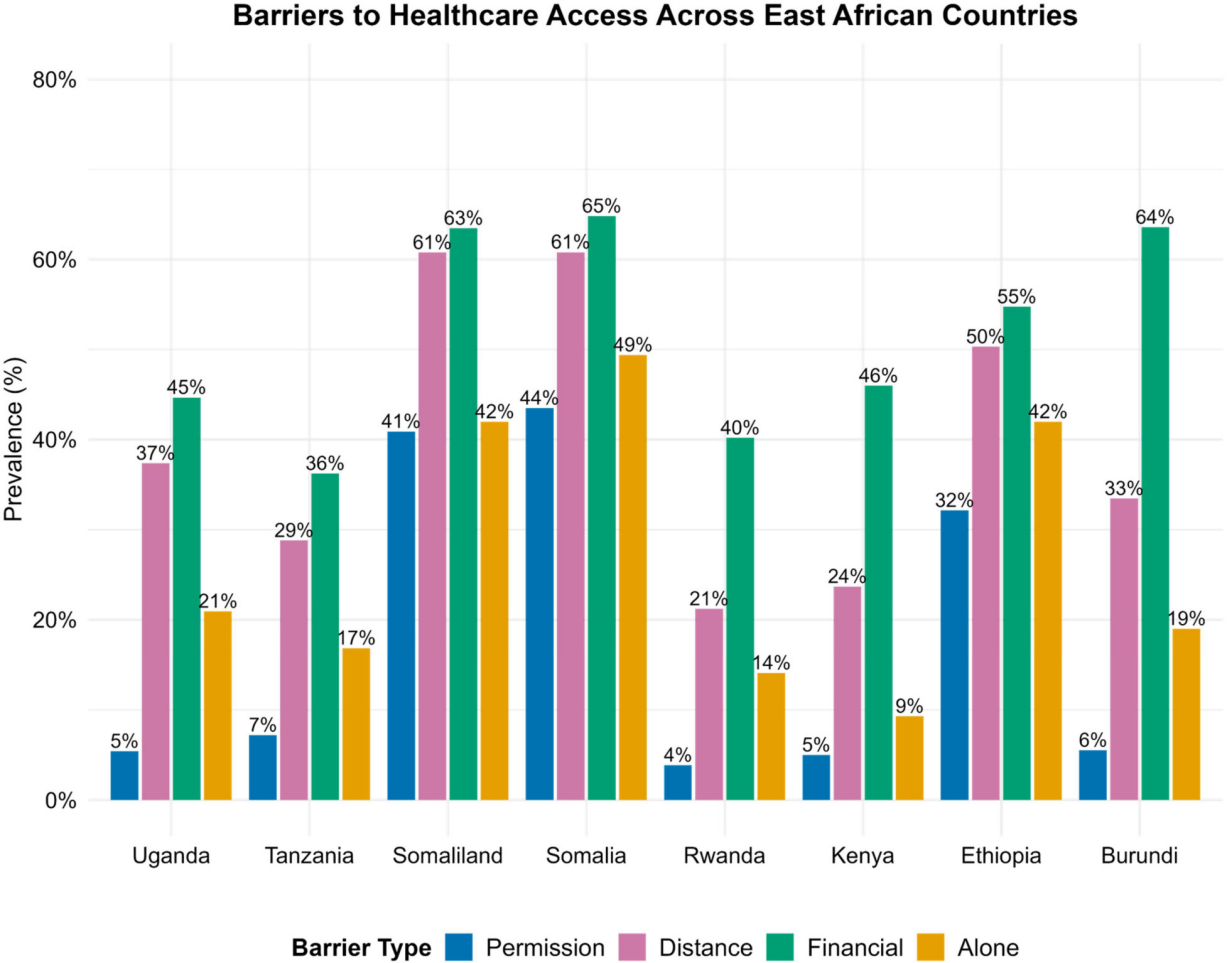
Barriers to healthcare access.

Figure 1 presents a cross-country comparison of key barriers to healthcare access among women in eight East African countries, as reported in the Demographic and Health Surveys (DHS). Four main barriers are shown:

**Needing permission to go for treatment**—reflects gendered and sociocultural constraints, where women may require approval from male family members or elders before seeking care.
**Distance to health facilities**—indicates geographic and infrastructural barriers, often exacerbated in rural and remote areas.
**Money needed for treatment (financial barrier)**—captures perceived affordability, including direct medical costs, transportation, and lost income.
**Not wanting to go alone**—relates to social support, safety concerns, and cultural norms around mobility and companionship.

Across the region, substantial variation is observed in the relative importance of these barriers. In most countries, financial constraints represent the most prevalent obstacle. For instance, the share of women citing money as a major barrier is 65% in Somalia, 64% in Burundi, 63% in Somaliland, and 55% in Ethiopia, compared with lower proportions in relatively better-off countries such as Tanzania (36%) and Uganda (45%).

**Notably, Somalia, Somaliland, and Burundi exhibited the highest levels of financial barriers**. This disparity can be attributed to several contextual factors:

**Somalia & Somaliland:** Both face protracted fragility, weak governance, and underfunded health systems heavily reliant on out-of-pocket payments and external aid. High levels of poverty, limited financial inclusion, and low female literacy further restrict women's ability to pay for care.

**Burundi:** Despite relative stability, Burundi struggles with extreme poverty, low public health expenditure, and a high dependency on out-of-pocket spending. Rural women in particular face compounded barriers due to geographic isolation and economic marginalization.

Barriers related to **distance to health facilities** are also widespread—reported by nearly 61% of women in Somalia and Somaliland and 50% in Ethiopia—reflecting persistent inequalities in rural infrastructure. In contrast, **social or cultural barriers**, such as reluctance to go alone for treatment, are the third most prevalent type of barrier, affecting 40%–50% of women in Somalia, Somaliland, and Ethiopia, compared with much lower levels in the other countries. Finally, **needing permission to seek care** is less common overall but remains pronounced in certain contexts; for example, the prevalence of this barrier is 44% in Somalia, 41% in Somaliland, and 32% in Ethiopia, compared with less than 10% in Kenya, Burundi, Tanzania, Uganda, and Rwanda.

Given this regional pattern, the subsequent analysis focuses specifically on the financial barrier (“money needed for treatment”), as it consistently emerges as the most widespread and quantifiable predictor of limited healthcare access across East Africa. Moreover, financial barriers correlate with structural and policy-relevant factors such as poverty, income inequality, and healthcare financing, whereas other barriers—such as permission or distance—are more influenced by cultural and geographic contexts that vary substantially across countries.

### Prevalence of financial barrier

As shown in Error! Reference source not found., the pooled prevalence of financial barrier to healthcare access across the eight East African countries is 49.7% (95% CI: 49.4%–50.0%), indicating that nearly half of women in the region report “money needed for treatment” as a major obstacle to seeking healthcare.

**Figure 2:**
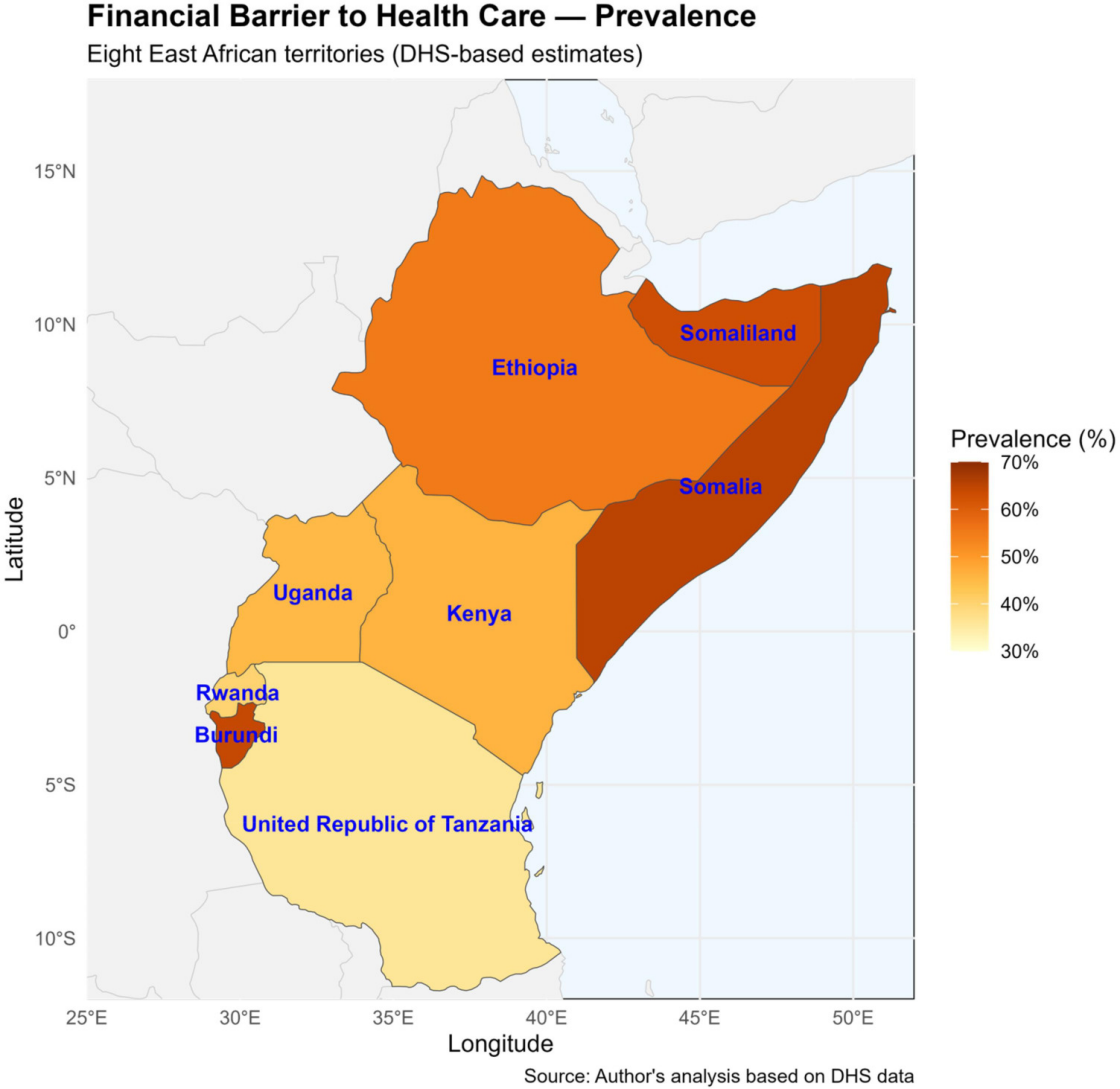
Spatial distribution of financial barrier.

As shown in Figure 2, substantial cross-country variation is observed. The highest prevalence of financial barriers was reported in Somalia (64.8%, 95% CI: 62.4–67.2) and Somaliland (63.5%, 95% CI: 62.4–67.2), followed closely by Burundi (63.6%, 95% CI: 62.1–65.0) and Ethiopia (54.8%, 95% CI: 51.9–57.5). In contrast, Tanzania exhibited the lowest prevalence (36.2%, 95% CI: 34.6–37.9), followed by Rwanda (40.2%, 95% CI: 38.6–41.8), Uganda (44.7%, 95% CI: 43.0–46.3), and Kenya (46.0%, 95% CI: 44.4–47.5). These disparities suggest that while financial barriers remain a pervasive obstacle across the region, their magnitude differs considerably.

### Disparities of financial barrier across place of residence and education level

**Figure 3:**
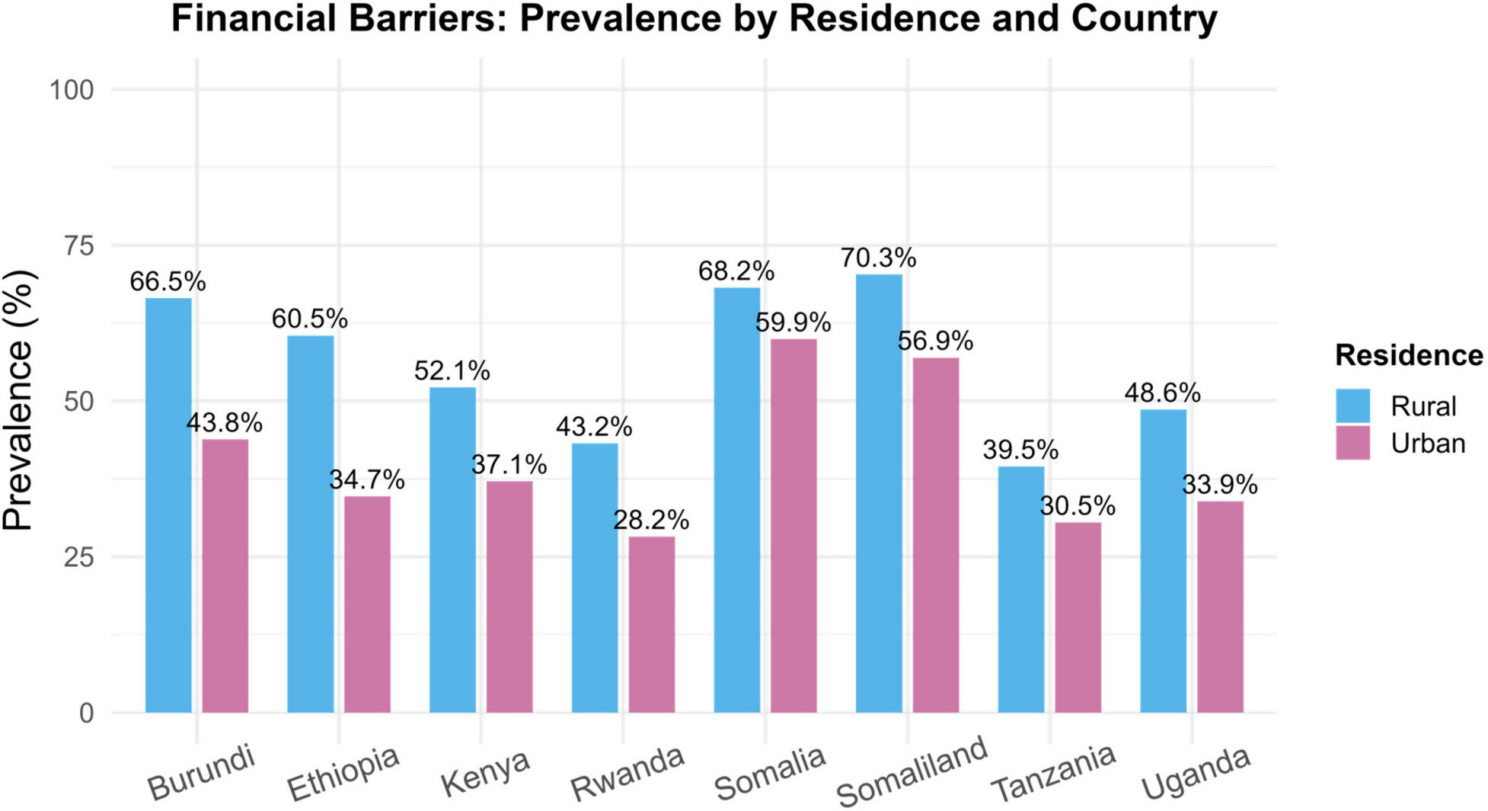
Financial barrier prevalence by residence type.

**Figure 4:**
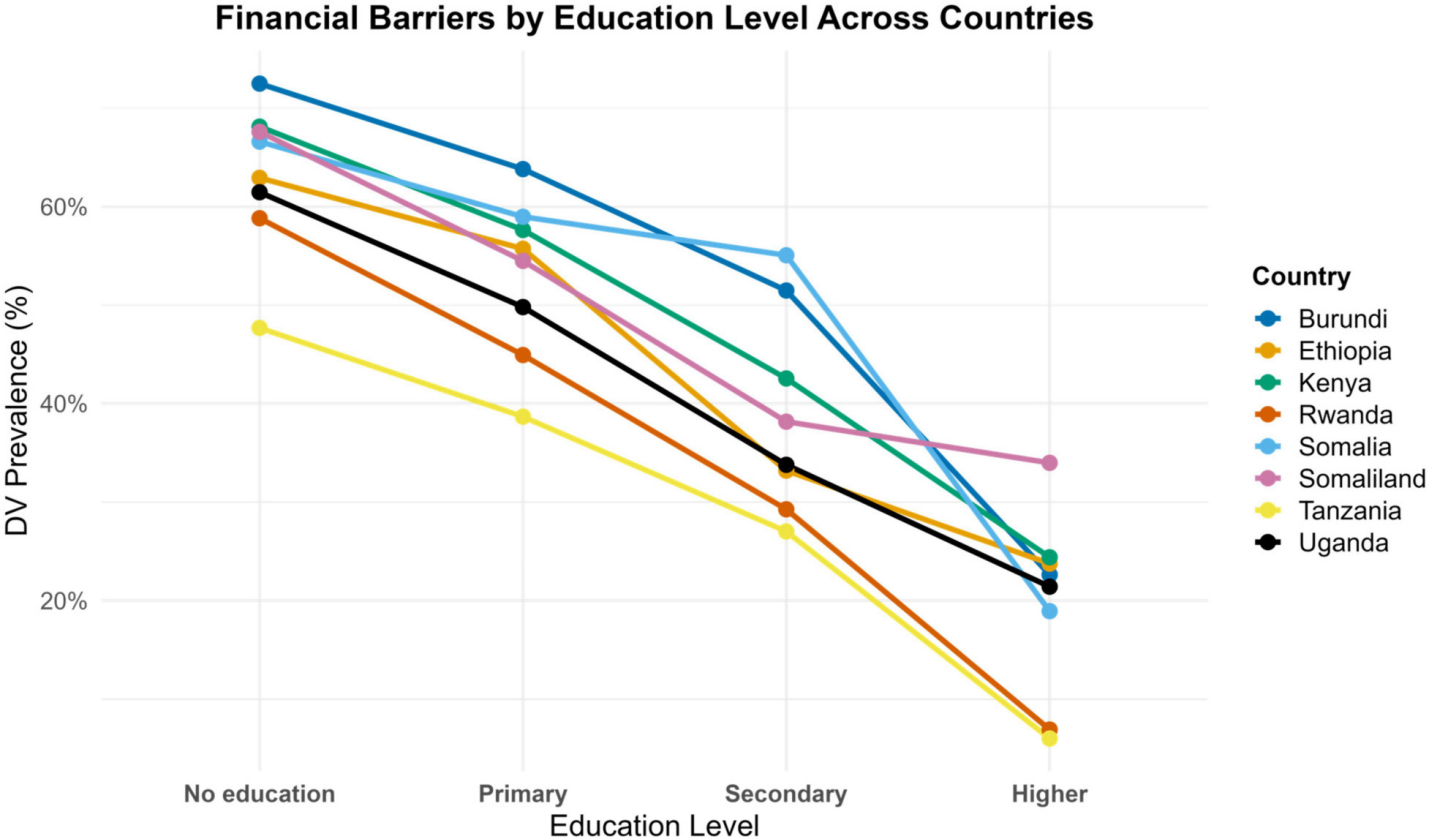
Financial barrier prevalence by education.

As shown in Figure 3, notable rural–urban disparities emerged in the prevalence of financial barrier to healthcare access among women (Table 2). Overall, rural women consistently reported higher levels of financial barrier compared to their urban counterparts, highlighting a persistent inequality in healthcare accessibility. The largest rural–urban gaps were observed in Somaliland (70.3% rural vs. 56.9% urban) and Somalia (68.2% vs. 59.9%), suggesting that women in rural areas of these contexts face particularly acute economic obstacles when seeking medical care. Similarly, marked disparities were evident in Burundi (66.5% vs. 43.8%) and Ethiopia (60.5% vs. 34.7%), where financial constraints appear intertwined with geographic disadvantage. In contrast, Rwanda (43.2% vs. 28.2%) and Tanzania (39.5% vs. 30.5%) exhibited relatively narrower but still meaningful rural–urban differences. The smallest gap was found in Uganda (48.6% vs. 33.9%) and Kenya (52.1% vs. 37.1%). In addition, as shown in Figure 4, among women with no education, the prevalence of financial barrier is highest, reaching 72.5% in Burundi, 68.1% in Kenya, and 67.6% in Somaliland. In contrast, the proportion drops sharply among women with higher education, falling below 25% in most countries, with the lowest rates observed in Tanzania (6.0%) and Rwanda (7.0%).

**Table 2:** Descriptive statistics.

Weighted sample size (*n*) = 108,175	Weighted frequency (%)	Has financial barrier		*P*-Value
Variable	Levels	Frequency (%)		
Country	Burundi	17,269 (15.96)	10,978 (63.57)	0.0000
	Ethiopia	15,683 (14.5)	8,588 (54.76)	
	Kenya	16,716 (15.45)	7,687 (45.98)	
	Rwanda	14,634 (13.53)	5,882 (40.19)	
	Somalia	6,633 (6.13)	4,299 (64.82)	
	Somaliland	3,480 (3.22)	2,209 (63.47)	
	Tanzania	15,254 (14.1)	5,529 (36.25)	
	Uganda	18,506 (17.11)	8,267 (44.67)	
Place of residence	Urban	30,325 (28.03)	11,502 (37.93)	0.0000
	Rural	77,851 (71.97)	41,938 (53.87)	
Women's age	15–19	21,845 (20.19)	9,773 (44.74)	0.0000
	20–24	19,910 (18.41)	9,177 (46.09)	
	25–29	18,963 (17.53)	9,416 (49.66)	
	30–34	15,753 (14.56)	8,078 (51.28)	
	35–39	13,761 (12.72)	7,253 (52.71)	
	40–44	10,057 (9.3)	5,396 (53.66)	
	45–49	7,886 (7.29)	4,347 (55.13)	
Women's education level	No education	28,560 (26.4)	18,492 (64.75)	0.0000
	Primary	46,954 (43.41)	23,912 (50.93)	
	Secondary	25,899 (23.94)	9,586 (37.01)	
	Higher	6,762 (6.25)	1,449 (21.43)	
Marital status	Never Married	30,095 (27.82)	13,072 (43.44)	0.0000
	Married/Living with Partner	65,972 (60.99)	33,099 (50.17)	
	Divorced/Separated	8,867 (8.2)	5,099 (57.50)	
	Widowed	3,241 (3)	2,170 (66.95)	
Women's work status	No	48,268 (44.62)	24,381 (50.51)	0.0004
	Yes	59,907 (55.38)	29,058 (48.51)	
Use of mobile for financial transactions	No	68,889 (63.68)	37,018 (53.74)	0.0000
	Yes	39,287 (36.32)	16,421 (41.80)	
Ownership of bank account	No	93,162 (86.12)	48,969 (52.56)	0.0000
	Yes	15,013 (13.88)	4,471 (29.78)	0.0000
Wealth index	Poorest	18,947 (17.52)	13,051 (68.88)	
	Poorer	19,866 (18.36)	11,890 (59.85)	
	Middle	20,492 (18.94)	10,670 (44.75)	
	Richer	22,332 (20.64)	9,994 (29.53)	
	Richest	26,538 (24.53)	7,836 (49.40)	

### Descriptive statistics of study participants

Table 2 presents the weighted distribution of the study sample and the prevalence of financial barrier to healthcare access among women in eight East African countries. The analysis is based on a weighted sample of 108,175 women. The largest proportion of participants were from Uganda (17.1%), followed by Burundi (16.0%), Kenya (15.5%), and Ethiopia (14.5%), while the smallest share came from Somaliland (3.2%). Most women resided in rural areas (72.0%), and the majority were aged 15–29 years (56.1%). Nearly 43% had primary education, 26% had no education, and only 6% attained higher education. Around 61% were married or living with a partner, while 28% were never married. More than half of the women (55%) were employed, although only 36% reported using mobile phones for financial transactions and 14% owned a bank account. Regarding economic status, 18% were in the poorest quintile and 25% in the richest. Bivariate results reveal substantial cross-country variation in the prevalence of financial barrier. The highest levels were observed in Somalia (64.8%), Burundi (63.6%), and Somaliland (63.5%), compared with lower rates in Tanzania (36.3%) and Rwanda (40.2%). Financial barrier was more common among rural residents (53.9%) than urban residents (37.9%). The prevalence increased slightly with age, from 44.7% among women aged 15–19 to 55.1% among those aged 45–49. Education showed a strong inverse relationship, declining from 64.8% among women with no education to 21.4% among those with higher education. Similarly, financial barrier was more prevalent among widowed (67.0%) and divorced/separated (57.5%) women than among married or cohabiting (50.2%) women. Women not engaged in paid work (50.5%), without access to mobile financial services (53.7%), or without a bank account (52.6%) were more likely to report financial barrier. The prevalence also decreased steadily across wealth quintiles, from 68.9% in the poorest to 29.5% in the richest. All associations were statistically significant (*p* < .001), indicating that financial barrier is strongly linked to socioeconomic and demographic disadvantage among women in East Africa.

### Inequalities across countries

**Figure 5:**
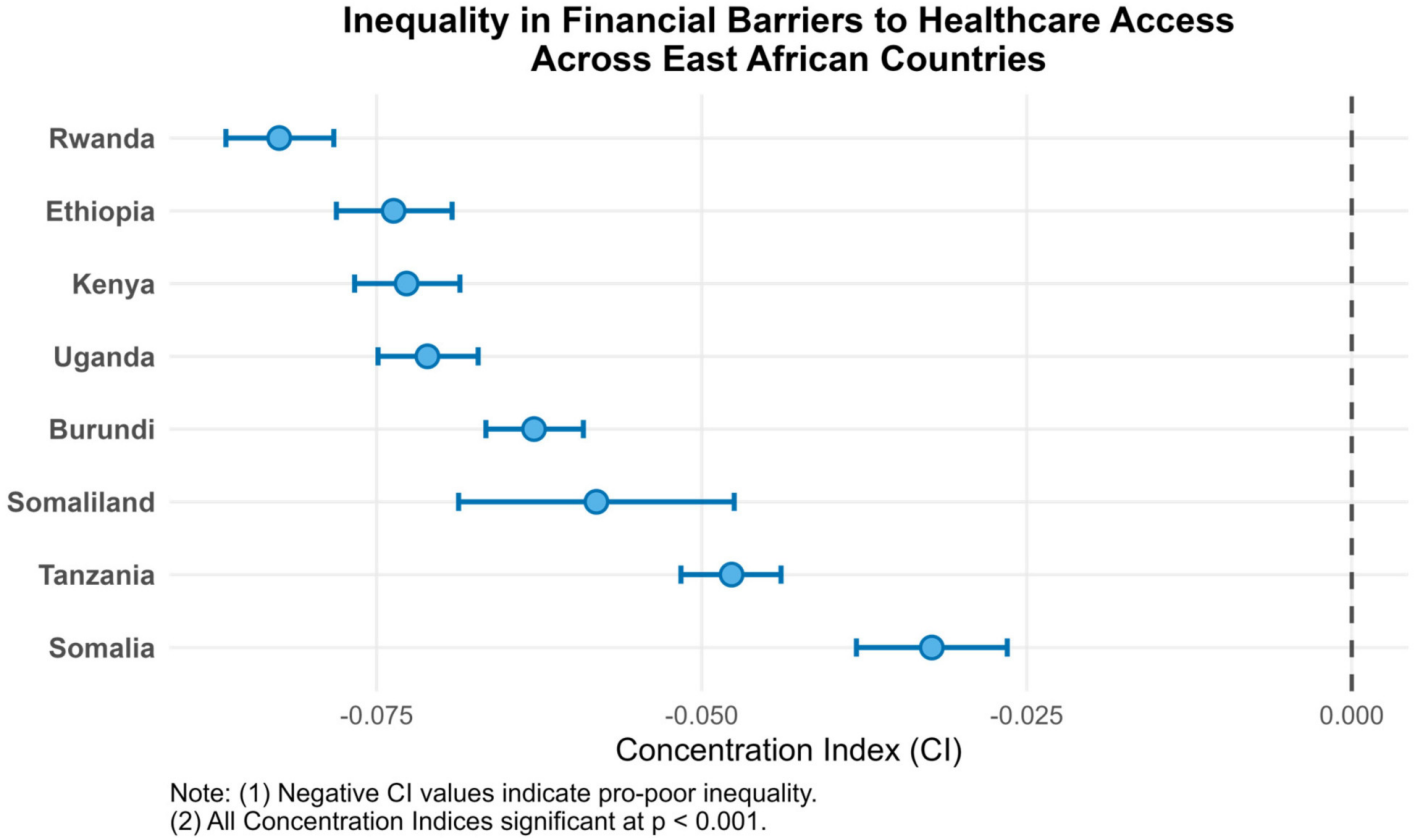
Concentration index.

Across all eight East African countries, the Concentration Index (CI) values were negative and statistically significant (*p* < .001) as shown Figure 5, indicating that financial barriers to healthcare are disproportionately concentrated among poorer households. The degree of inequality varied notably across countries, with the highest pro-poor concentration observed in Rwanda (CI = –0.0825), followed by Ethiopia (CI = –0.0737), Kenya (CI = –0.0727), and Uganda (CI = –0.0711). Moderate inequalities were evident in Burundi (CI = –0.0629) and Somaliland (CI = –0.0581), while Tanzania (CI = –0.0477) and Somalia (CI = –0.0323) exhibited comparatively smaller disparities.

### Multilevel binary logistic regression result

In the empty model (Model 0), the total variance attributed to higher levels was 0.42, with an intra-class correlation (ICC) of 11.32%. Decomposition of this variance indicated that approximately 7.1% (ICC household) of the total variance resided at the household level and 4.2% (ICC cluster) at the cluster/community level, justifying the use of a three-level structure.

Table 3 presents the results of the multilevel analysis for financial barrier to healthcare access in East Africa. The empty model (Model 0) indicated significant clustering at the household and community levels (ICC = 11.3%). Successive models including individual- and household-level factors (Model I) and country- and residence-level factors (Model II) improved model fit and reduced unexplained variance. The full model (Model III), which included all individual, household, and contextual factors, demonstrated the lowest AIC (134,377) and BIC (134,656), the highest log-likelihood (−67,160), and the smallest residual variance (0.20; ICC = 5.8%). These results indicate that Model III provides the most comprehensive and parsimonious explanation of financial barrier across clusters. Accordingly, all further interpretations are based on Model III.

**Table 3:** Multivariable multilevel binary logistic regression results for financial barriers to healthcare access. Model III (final full model) is the primary basis for interpretation.

Variable	Empty model	Model I AOR (95% CI)	Model II AOR (95% CI)	Model III AOR (95% CI)
Country				
Burundi			1 (Ref)	
Ethiopia			0.66 (0.63–0.70)*	0.60 (0.57–0.64)*
Kenya			0.69 (0.66–0.73)*	0.59 (0.56–0.63)*
Rwanda			0.39 (0.37–0.40)*	0.37 (0.35–0.39)*
Somalia			1.39 (1.30–1.50)*	0.80 (0.73–0.87)*
Somaliland			1.45 (1.32–1.58)*	0.90 (0.81–0.99)*
Tanzania			0.32 (0.31–0.34)*	0.27 (0.26–0.29)*
Uganda			0.53 (0.51–0.56)*	0.49 (0.47–0.51)*
Place of residence				
Urban			1 (Ref)	
Rural			2.01 (1.94–2.07)*	0.84 (0.80–0.87)*
Women's age				
15–19		1 (Ref)		
20–24		1.24 (1.18–1.29)*		1.22 (1.16–1.28)*
25–29		1.42 (1.35–1.49)*		1.42 (1.34–1.49)*
30–34		1.42 (1.34–1.50)*		1.46 (1.38–1.54)*
35–39		1.46 (1.38–1.54)*		1.54 (1.45–1.63)*
40–44		1.47 (1.38–1.56)*		1.59 (1.49–1.69)*
45–49		1.49 (1.40–1.59)*		1.68 (1.57–1.80)*
Education level				
No education		1 (Ref)		
Primary		0.66 (0.64–0.68)*		0.86 (0.83–0.89)*
Secondary		0.51 (0.49–0.53)*		0.66 (0.63–0.69)*
Higher		0.35 (0.32–0.37)*		0.41 (0.38–0.45)*
Marital status				
Never married		1 (Ref)		
Married/living with partner		0.78 (0.75–0.81)*		0.77 (0.74–0.80)*
Divorced/separated		1.20 (1.13–1.27)*		1.22 (1.15–1.30)*
Widowed		1.31 (1.20–1.43)*		1.22 (1.12–1.34)*
Work status				
No		1 (Ref)		
Yes		0.99 (0.96–1.01)		0.99 (0.96–1.02)
Mobile money use				
No		1 (Ref)		
Yes		0.88 (0.85–0.91)*		1.00 (0.96–1.04)
Bank account				
No		1 (Ref)		
Yes		0.69 (0.66–0.73)*		0.69 (0.66–0.73)*
Wealth index				
Poorest		1 (Ref)		
Poorer		0.72 (0.69–0.75)*		0.69 (0.66–0.72)*
Middle		0.55 (0.52–0.57)*		0.51 (0.48–0.53)*
Richer		0.43 (0.41–0.45)*		0.37 (0.36–0.39)*
Richest		0.28 (0.26–0.29)*		0.21 (0.20–0.22)*
Evaluation metrics				
AIC	148,369	137,652	142,577	134,377
BIC	148,388	137,854	142,673	134,656
Loglikelihood	−74,183	−68,805	−71,278	−67,160
Variance	0.42	0.23	0.28	0.20
ICC	11.32%	6.49%	7.74%	5.83%

*Indicates statistical significance at *p* < 0.05.

In the full multilevel model (Model III), several individual, household, and contextual factors were significantly associated with financial barrier to healthcare access. Compared to Burundi, women in Ethiopia (AOR = 0.60, 95% CI: 0.57–0.64), Kenya (0.59, 0.56–0.63), Rwanda (AOR = 0.37, 0.35–0.39), Tanzania (AOR = 0.27, 0.26–0.29), Uganda (AOR = 0.49, 95% CI: 0.47–0.51), Somalia (AOR = 0.80, 95% CI: 0.73–0.87), and Somaliland (AOR = 0.90, 95% CI: 0.81–0.99) had lower odds of experiencing financial barrier. Contrary to the model II findings, the adjusted model showed that rural women had lower odds of financial barriers than urban women (AOR=0.84). This association reflects a comparison among women of similar wealth, education, and other socioeconomic characteristics. It indicates that when poverty is accounted for, rural residence itself was not associated with higher odds of perceived financial barriers in the adjusted model and may even confer a slight contextual advantage. This reversal reflects Simpson's Paradox: the strong confounding effect of the higher poverty and lower education concentrated in rural areas masked a modest protective effect of rural residence itself, which may stem from lower costs or stronger community support. In addition, older women faced higher odds, with those aged 45–49 years having 1.68 times higher odds than women aged 15–19 years (AOR = 1.68, 95% CI: 1.57–1.80). Higher educational attainment was associated with 59% lower odds compared to those with no education (AOR = 0.41, 95% CI: 0.38–0.45). Marital status was also important: married women had lower odds (AOR = 0.77, 95% CI: 0.74–0.80), while divorced/separated (AOR = 1.22, 95% CI: 1.15–1.30) and widowed women (1.22, 95% CI: 1.12–1.34) had higher odds. Ownership of a bank account was associated with reduced odds (AOR = 0.69, 95% CI: 0.66–0.73). In addition, Wealth showed a clear gradient, with the richest women 79% less likely to experience financial barrier than the poorest (AOR = 0.21, 95% CI: 0.20–0.22). whereas use of mobile money for financial transaction and employment status was not significant in Model III.

## Discussion

This study provides a comprehensive analysis of financial barriers to healthcare access among women of reproductive age in eight East African countries, utilizing recent, nationally representative Demographic and Health Surveys (DHS) pooled with appropriate weighting adjustments. The findings reveal that perceived financial constraints are the most pervasive and significant self-reported obstacle to healthcare access in the region, affecting nearly half (49.7%) of all women surveyed. This prevalence is substantially higher than other barriers, such as distance to facilities or the need for permission to seek care, underscoring that the affordability of services, rather than just their physical availability, is the primary challenge.

The profound disparities observed are a central finding of this study. The concentration indices, which were negative and statistically significant across all countries, provide robust quantitative evidence that financial barriers are disproportionately concentrated among the poor, a clear manifestation of socioeconomic inequality in healthcare access (38, 39). This economic gradient is further corroborated by the strong association between lower wealth quintiles, lower educational attainment, and higher odds of experiencing financial barriers. The stepwise reduction in odds from the poorest to the richest wealth quintile, culminating in a 79% lower odds for the richest women, illustrates a steep social gradient in the ability to pay for healthcare. This finding aligns with the well-established literature on health disparities, which consistently identifies socioeconomic status as a fundamental correlate of access to care (38, 39). The association of higher education and financial inclusion with lower odds (bank account ownership) further emphasizes that empowerment and economic resources are critical in mitigating financial barriers (41–43).

These financial barriers are particularly acute for women of reproductive age from the most disadvantaged segments—those who are poorest, rural, and with little or no formal education. This intersection of gender, poverty, and geography creates a triple burden that severely limits access to essential maternal and reproductive healthcare. Poor women often lack financial autonomy, depend on irregular incomes, and face higher opportunity costs when seeking care—such as lost wages or childcare responsibilities—which magnifies the perceived and real cost of healthcare (26, 44). Additionally, women in rural areas not only travel farther to facilities but also have fewer alternative funding sources, such as community savings groups or mobile banking services, further entrenching their financial exclusion.

While financial barriers emerged as the most pervasive obstacle across the region, our analysis reveals distinct interactions between financial, permission, distance, and social barriers that vary by national context. In countries like Somalia and Somaliland, high financial barriers are compounded by significant challenges related to distance and the need for permission, reflecting a convergence of infrastructural deficit, socioeconomic fragility, and restrictive gender norms. In contrast, in Burundi**,** extreme poverty drives high financial barriers, while permission-related constraints are relatively low. More stable systems such as Rwanda and Tanzania exhibit lower overall barriers, yet rural women still face a combination of financial and geographic challenges. This interplay suggests that while financial access is the foundational and most widespread challenge, effective interventions must be multifaceted, addressing the specific secondary barriers—whether geographical, cultural, or social—that exacerbate financial exclusion in each setting.

Our multilevel analysis offers nuanced insights into the factors associated with financial barriers. The significant intra-class correlation (ICC) in the null model confirmed that financial barriers are clustered within households and communities, justifying the use of a multilevel modeling approach. The full model, which included individual, household, and contextual factors, provided the best fit, explaining a substantial portion of the variance and reducing the ICC from 11.32% to 5.83%. This indicates that while contextual factors matter, a significant share of the variance in financial barriers is explained by individual and household-level socioeconomic characteristics.

A critical and intriguing finding was the reversal of the association between rural residence and financial barriers after adjusting for confounders. In the bivariate and Model II analyses, rural women appeared to have higher odds of financial barriers. However, in the full adjusted model (Model III), rural residence was associated with a 16% reduction in odds compared to urban residence. This phenomenon, a classic example of Simpson's Paradox, reveals that the raw disadvantage observed in rural areas is primarily explained by the confounding effects of higher poverty and lower education levels concentrated in those areas. Once these socioeconomic factors are controlled for, the analysis suggests that the analysis suggests an adjusted association between rural residency and a modestly lower likelihood of reporting a financial barrier. This adjusted protective effect could be hypothesized to stem from factors such as potentially lower costs for basic services at public health centers in rural areas, stronger informal community support networks that help absorb healthcare costs, or different healthcare-seeking patterns where formal care is sought only for severe conditions, thereby reducing perceived financial stress for routine care (45). It is crucial to emphasize that this finding represents a comparison holding socioeconomic status constant; in reality, because poverty is highly concentrated in rural areas, rural women continue to face high absolute rates of financial barriers. The key policy insight is that the primary driver of financial access problems is poverty, not rurality *per se*.

The stark cross-country variations in the prevalence of financial barriers, from 64.8% in Somalia to 36.2% in Tanzania, highlight the critical role of national health financing policies and systems. Countries like Rwanda and Tanzania, which have made significant strides towards Universal Health Coverage (UHC) through robust community-based health insurance schemes and increased public health spending (21, 25), are associated with lower levels of financial hardship. Conversely, the high prevalence in countries like Somalia, Somaliland, and Burundi reflects the severe challenges of fragile health systems, underfunding, and a heavy reliance on out-of-pocket (OOP) payments (21, 31). The heavy dependence on OOP financing, documented in our background and evidenced by the high barrier prevalence, is widely recognized as an inequitable model that deters service use and can plunge households into poverty (8, 30, 32), directly undermining the goal of UHC.

Our study situates these financial barriers within the broader context of maternal health outcomes. The World Bank estimates that 74% of maternal deaths could be prevented with full access to interventions, particularly emergency obstetric care (46). The high financial barriers identified in this study directly compromise this access. When women cannot afford treatment, they are more likely to miss or delay essential care during pregnancy, childbirth, and the postnatal period, leading to the preventable maternal deaths that continue to plague the region, with Sub-Saharan Africa accounting for approximately two-thirds of the global burden (19, 28). Therefore, addressing financial barriers is not merely a matter of economic policy but an urgent imperative for saving lives and achieving the Sustainable Development Goals (6, 20).

### Policy implications

The compelling evidence generated by this study outlines several critical pathways for policy intervention. Our findings show clear linkages that should inform targeted actions.

For policymakers across the eight East African countries, these findings underscore several urgent priorities: implement pro-poor, gender-sensitive financing mechanisms such as fee exemptions for maternal care and subsidized insurance; accelerate financial inclusion by expanding women's access to bank accounts and mobile money; strengthen rural health infrastructure through mobile clinics and community health workers to reduce indirect costs; leverage cross-sectoral partnerships to advance female education and economic empowerment; and tailor strategies to national contexts**,** prioritizing humanitarian safety nets and community-based health insurance in fragile settings like Somalia and Somaliland, while scaling proven insurance models in more stable systems such as Rwanda and Tanzania. In high-burden settings such as Somalia, Somaliland, and Burundi, where fragile health systems and poverty intensify financial barriers, policy efforts should prioritize humanitarian health financing, expanded fee-waiver programs for maternal care, and the integration of community-based health insurance within stable regions to protect households from catastrophic expenditures. Without such equity-focused, multi-sectoral interventions, financial barriers will continue to perpetuate cycles of poor maternal health and intergenerational poverty across the region.

Primarily, our finding that women without bank accounts face higher odds of financial barriers supports the recommendation to promote financial inclusion as part of broader health access strategies. Addressing a challenge as multifaceted as financial access to healthcare also demands concerted multi-sectoral action. Interventions confined solely to the health sector will be insufficient. Broader policies aimed at poverty reduction, advancing female education, and fostering economic empowerment—for instance, by promoting financial inclusion through bank account ownership—will have synergistic effects. Empowering women economically and educating them represent important long-term strategies for addressing the socioeconomic roots of health inequities.

Furthermore, given the concentrated burden of financial barriers among the most disadvantaged segments of the population, policies must be explicitly pro-poor to effectively address these inequities. Targeted interventions are essential and should include measures such as providing fee exemptions for specific services, implementing conditional or unconditional cash transfers, and subsidizing health insurance premiums for vulnerable groups. The objective of such targeted approaches is to reduce the likelihood that cost becomes a deterrent to seeking essential healthcare for the poorest, least educated, and those in rural areas.

Consistent with evidence from Rwanda and Tanzania (21, 25), our results underscore the potential value of expanding social health insurance schemes or strengthened tax-funded systems. East African governments should prioritize shifting health financing away from a reliance on inequitable out-of-pocket payments by scaling up pre-payment and risk-pooling mechanisms. This can be achieved through the expansion of social health insurance schemes or strengthened tax-funded systems, drawing inspiration from the documented successes of regional leaders like Rwanda and Tanzania (21, 25). Finally, the stark disparities observed across the region underscore that a one-size-fits-all approach is inadequate. In fragile contexts such as Somalia and Somaliland, where financial barriers are most acute, tailored strategies are urgently required. This includes mobilizing targeted international support and developing innovative financing mechanisms to build resilient and adequately funded health systems. This includes mobilizing targeted international aid for health system strengthening, piloting community-based health insurance in stable regions, and implementing explicit pro-poor fee-waiver policies. The goal in these settings must be to shield households from catastrophic health expenditures that deepen poverty and perpetuate poor health outcomes.

## Limitations

Notwithstanding its contributions, this study is subject to several limitations. First, its cross-sectional design, inherent in the DHS data, precludes the establishment of causal relationships. Second, our outcome variable is a self-reported, binary measure of perceived financial difficulty. While this perception is a powerful determinant of health-seeking behavior and is widely used in comparative studies, it is a subjective proxy for financial barriers. While the self-reported nature of the outcome variable introduces the possibility of perceptual bias, it remains a validated and widely used proxy for affordability constraints in cross-country health surveys, and it directly reflects the user perspective that shapes care-seeking decisions. It may not fully align with objective measures of financial hardship, such as catastrophic health expenditure, and the dichotomous “big problem” vs. “not a big problem” response may obscure variations in the severity of financial stress. Nevertheless, it provides a standardized, patient-centered perspective on affordability across the eight countries.

Third, the analysis was constrained by the absence of potentially relevant variables in the datasets, such as detailed information on the quality of care or specific health insurance coverage. Fourth, geographically, the study's scope was limited to eight East African countries for which recent DHS data were available. Neighboring nations in the region, namely Djibouti, South Sudan, and Eritrea, were excluded due to the outdated nature or absence of their DHS datasets. This exclusion was necessary to ensure reliability and temporal comparability but means the findings may not fully represent the entire East African region. Furthermore, while the patterns observed are consistent with broader literature on financial access in LMICs, caution is warranted in generalizing specific results—such as the rural-urban disparity reversal—to dissimilar health system or socioeconomic contexts outside the studied countries.

Fifth, the DHS surveys used were conducted between 2016 and 2022. Although we selected the most recent available survey for each country, temporal differences could introduce heterogeneity, as economic conditions, health policies, or health-seeking behaviors may have changed within this period. However, to our knowledge, no major health financing reforms were implemented in these countries during this interval that would fundamentally alter the cross-sectional pattern of financial barriers observed here.

Sixth, while this study provides a detailed regional analysis of financial barriers in eight East African countries, its generalizability should be interpreted with care. Our findings are most directly applicable to the studied countries and reflect the period 2016–2022. Financial barriers to healthcare are widely recognized as a critical challenge across low- and middle-income countries (LMICs), particularly in sub-Saharan Africa where out-of-pocket payments remain high (8, 30). However, the specific magnitude of inequality and the adjusted association between rural residence and lower odds of financial barriers may vary depending on local health system structures, economic conditions, and cultural contexts. For example, the success of community-based health insurance in Rwanda or expanded public financing in Tanzania may not be directly replicable in countries with weaker governance or ongoing conflict. Therefore, while our results underscore a common regional challenge and highlight important equity-oriented policy directions, their direct application to other settings should consider contextual differences in health financing, poverty profiles, and health service delivery.

## Conclusion

In conclusion, this study demonstrates that financial barriers are a predominant and inequitable obstacle to healthcare access for women in the eight studied East African countries. These barriers are deeply rooted in socioeconomic disadvantage and are exacerbated by health financing systems that rely heavily on out-of-pocket payments. The findings provide a robust evidence base for policymakers in the region, highlighting the urgent need to accelerate health financing reforms aimed at advancing Universal Health Coverage. Implementing pro-poor, equity-oriented policies that reduce financial barriers—informed by evidence from countries like Rwanda and Tanzania—could help East African nations move closer to the goal of ensuring that all women, regardless of economic status or residence, are able to access quality healthcare without undue financial hardship.

## Data Availability

The data used in this study are available from the Demographic and Health Surveys (DHS) Program website https://dhsprogram.com/data upon reasonable request. Access to the data requires free registration and approval from the DHS Program. The authors obtained permission to use the data through the DHS Program’s data request system.
